# Systems genetics of obesity in an F2 pig model by genome-wide association, genetic network, and pathway analyses

**DOI:** 10.3389/fgene.2014.00214

**Published:** 2014-07-09

**Authors:** Lisette J. A. Kogelman, Sameer D. Pant, Merete Fredholm, Haja N. Kadarmideen

**Affiliations:** Animal Genetics, Bioinformatics and Breeding Section, Department of Veterinary Clinical and Animal Sciences, Faculty of Health and Medical Sciences, University of CopenhagenCopenhagen, Denmark

**Keywords:** obesity index, animal model, high-throughput genotype data, systems genetics, WISH network

## Abstract

Obesity is a complex condition with world-wide exponentially rising prevalence rates, linked with severe diseases like Type 2 Diabetes. Economic and welfare consequences have led to a raised interest in a better understanding of the biological and genetic background. To date, whole genome investigations focusing on single genetic variants have achieved limited success, and the importance of including genetic interactions is becoming evident. Here, the aim was to perform an integrative genomic analysis in an F2 pig resource population that was constructed with an aim to maximize genetic variation of obesity-related phenotypes and genotyped using the 60K SNP chip. Firstly, Genome Wide Association (GWA) analysis was performed on the Obesity Index to locate candidate genomic regions that were further validated using combined Linkage Disequilibrium Linkage Analysis and investigated by evaluation of haplotype blocks. We built Weighted Interaction SNP Hub (WISH) and differentially wired (DW) networks using genotypic correlations amongst obesity-associated SNPs resulting from GWA analysis. GWA results and SNP modules detected by WISH and DW analyses were further investigated by functional enrichment analyses. The functional annotation of SNPs revealed several genes associated with obesity, e.g., *NPC2* and *OR4D10*. Moreover, gene enrichment analyses identified several significantly associated pathways, over and above the GWA study results, that may influence obesity and obesity related diseases, e.g., *metabolic processes.* WISH networks based on genotypic correlations allowed further identification of various gene ontology terms and pathways related to obesity and related traits, which were not identified by the GWA study. In conclusion, this is the first study to develop a (genetic) obesity index and employ systems genetics in a porcine model to provide important insights into the complex genetic architecture associated with obesity and many biological pathways that underlie it.

## Introduction

Obesity, a complex condition characterized by excessive accumulation of body fat, is an exponentially growing public health problem associated with several severe diseases like Type 2 Diabetes, cardiovascular diseases, and various types of cancers (Bener et al., 2005). Obesity in humans is influenced by environmental, epigenetic, and genetic factors. Animal (model) studies show that weight gain and adiposity are related to genetic differences in eating behavioral patterns (Do et al., 2013), and it has also been shown that epigenetics play a critical role (e.g., nutritional status during pregnancy/fetal programming events) in determining effective partitioning of nutrients to maintaining essential physiological functions (Hou et al., 2013; Do et al., 2014). However, these environmental and epigenetic factors are difficult to manage, and therefore there is substantial interest in gaining more knowledge about the genetic background of obesity. Results from GWA studies performed on obesity-related traits, e.g., body mass index (BMI) and waist-hip ratio (WHR), are to date unsatisfactory in unraveling the genetic background for obesity. While phenotypes like BMI are known to be highly heritable (40–70%), the largest GWA study on BMI has only been able to identify 32 candidate loci that together explain only 1.45% of the inter-individual variation (Speliotes et al., 2010). Similarly in the case of WHR, only 14 candidate loci were identified that together explain only 1.03% of the variance in WHR compared to an estimated heritability between 22 and 61% (Heid et al., 2010). Beside gene-environment interactions (Qi and Cho, 2008), it is becoming increasingly evident that genome-wide genetic interactions, which are not taken into account by GWA studies, could play a key role to these discrepancies (Cordell, 2009). For example, the well-known obesity-related *FTO* gene interacts with *APOE* which in turn, is associated with Alzheimer's disease (Keller et al., 2011) and with *MC4R*, resulting in a higher chance of breast cancer (Cunha et al., 2013). These studies demonstrate both the presence of genetic interactions, and how these interactions could potentially influence the biological relationship between obesity and several other severe diseases. Therefore, the application of network-based genetics or systems genetics approaches directed toward investigating these genome-wide interactions and their functional relevance has the potential to provide novel biological and genetic insights into complex traits and diseases (Kadarmideen et al., 2006; Joshi et al., 2009; Civelek and Lusis, 2014; Kadarmideen, in press).

Various systems genetics approaches have been developed to unravel the genetic background of complex diseases by distinguishing networks, functional pathways, and underlying causal genes (Brazhnik et al., 2002; Segal et al., 2003; Diez et al., 2010; Horvath, 2011; Kadarmideen et al., 2011; Yang et al., 2011; Kadarmideen, in press). Recently, we published the Weighted Interaction SNP Hub (WISH) network method, which identifies clusters of highly interconnected SNPs (modules) using high throughput genotype data (Kogelman and Kadarmideen, 2014). Functional annotation and pathway analysis of detected modules may lead to the identification of biologically relevant pathways. Moreover, the differential connectivity or differential wiring of SNPs between two subgroups of extreme phenotypes, may reveal biologically interesting candidates as they might point to differences in underlying genetic regulation for manifestation of the trait of interest.

In the present study, we applied WISH network and gene enrichment analyses in addition to GWA analysis (validated using LDLA and haploblock methods), in order to identify individual variants and pathways that may be causal for obesity using a porcine model established for obesity studies (Kogelman et al., 2013). Animal models are helpful to study complex diseases due to reduced costs, more controlled environment, and the possibility of more controlled and extensive phenotyping in comparison with human studies. Pigs are a valuable model for human obesity and obesity-related diseases primarily because of comparable features that include a similar cardiovascular system, proportionally similar organ sizes, and comparable protein and lipid metabolism (Spurlock and Gabler, 2008). Pigs are also genetically close to humans and therefore acknowledged as an important biomedical model to study obesity (Groenen et al., 2012). The F2 pig resource population used in this study was constructed by intercrossing Göttingen Minipigs that are prone to obesity, and production pigs that are selected for leanness over many generations. This population has a high degree of genetic variation among several obesity- and obesity related traits (Kogelman et al., 2013) and is therefore a promising resource for further analyses directed toward identifying novel genetic determinants of obesity and obesity related diseases.

The main goal of this study was to investigate the genetic determination of obesity by applying integrated systems approaches including GWA, LDLA, WISH network analyses of whole genomic data and functional gene enrichment analyses, in a pig model. Here we report SNPs, and associated genes, biological pathways and functional ontologies identified via the application of these approaches to a combination of obesity phenotypes.

## Results

### Obesity index

**Figure 1:**
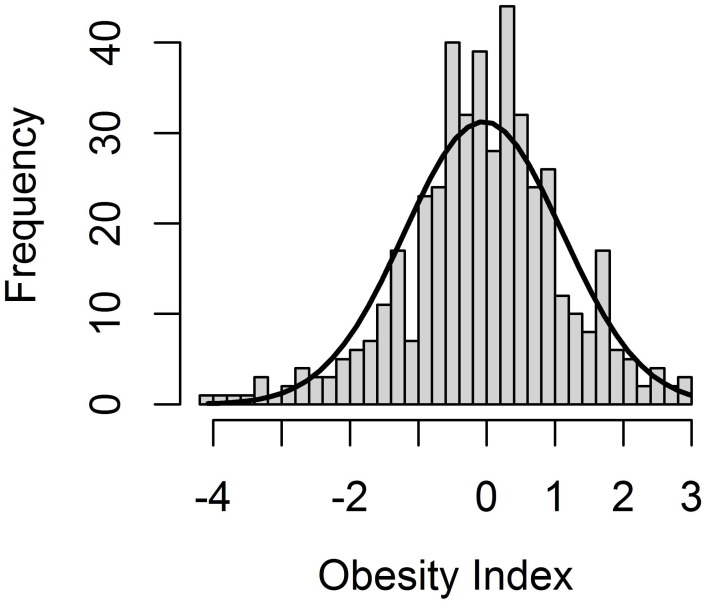
**Distribution of the Obesity Index (OI), an aggregate genotype representing the degree of obesity of all F2 animals in the F2 pig resource population**.

The Obesity Index (OI) was constructed according to the selection index theory (Cameron, 1997), whereby nine obesity and obesity-related (OOR) phenotypes are combined into a single value representative of the genetic predisposition to obesity. As expected based on the experimental design, the OI followed a normal distribution within the F2 pig resource population (Figure 1), with a mean of −0.06 and a standard deviation of 1.15. Accordingly, the OI can be used to select animals that are genetically predisposed to being either extremely obese or lean.

### Genome-wide association study (GWAS)

**Figure 2:**
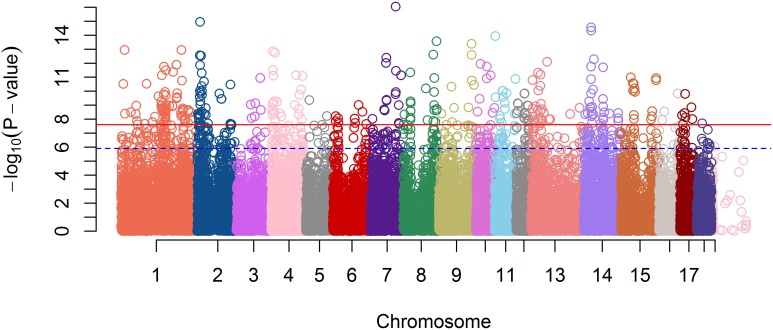
**A Manhattan plot of GWA Study single-locus *P*-values**. The blue dash line indicates a suggestive significance threshold with adjusted Bonferroni correction at *P*_adj_ = 1.33E^−6^ and the red line indicates a highly significant threshold with adjusted Bonferroni correction at *P*_adj_ = 2.67E^−8^.

A GWAS on the OI was performed on the entire F2 pig resource population. After quality control of the high-throughput genotype data, 40,910 markers and 538 animals were used for analysis. In total, 404 SNPs passed the Bonferroni corrected significance threshold of *P* = 2.44E^−8^, of which 366 were assigned to the porcine assembly, all located on autosomes. The Manhattan plot representing genome-wide *p*-values is shown in Figure 2.

In total, 289 genes were detected within 20 kb of the 366 SNPs that passed the genome-wide significance threshold. As most of the significantly associated SNPs were mapped to Ensemble gene identifiers (in total 100 unique genes), we only focused on those genes, and not on all the genes within a flanking distance of 20 Kb. Several of those genes could be associated with obesity or obesity-related diseases. Here we focus only on arbitrarily chosen seven genes corresponding to the highest genome-wide significant SNPs, detected after control of multiple testing and false discovery rate. Table 1 shows these SNP names, their genome-wide corrected *p*-values, their effects on total obesity score along with associated gene names.

**Table 1:** **Description of a selection of highly significant SNPs associated with the Obesity Index**.

**SNP**	**Chr**	**Position**	**Nearest gene**	**Effect**	***P*-value**
rs81396056	7	103574383	NPC2	0.63	9.09E^−17^
rs81238148	2	11449934	OR4D10	−0.60	1.13E^−15^
rs81416774	9	135204695	CACNA1E	−0.76	2.56E^−13^
rs80910848	1	228929456	SH3GL2	−0.49	1.31E^−12^
rs81306707	1	177369143	CDH20	0.50	1.79E^−12^
rs80998394	14	29824188	AACS	0.75	1.49E^−10^
rs80826774	7	65275011	ADPGK	0.76	4.20E^−10^

The most significant SNP (*P*-value = 9.09E^−17^) detected by the GWA study is located within the *NPC2* gene (Nieman–Pick disease, type 2C). This gene encodes a protein that plays a role in the regulation of cholesterol transport through the late endosomal/lysosomal system, affecting cholesterol homeostasis (Storch and Xu, 2009). The second highest significant SNP (*P*-value = 1.13E^−15^) is located approximately 3 Kb downstream from the *OR4D10* gene, which is an olfactory receptor gene. Olfactory receptors are responsible for the perception of smell, through neuronal responses, and thereby affecting the perception of food flavor. Furthermore, additional highly significant SNPs mapped within the vicinity of other potentially obesity related genes: e.g., *CACNA1E, SH3GL2, CDH20, AACS*, and *ADPGK.* The *CACNA1E* gene encodes a protein in a voltage dependent calcium channel. Variants in this gene have been associated with type 2 diabetes, insulin resistance, and impaired insulin secretion in non-diabetic subjects (Trombetta et al., 2012). The *SH3GL2* gene encodes Endophilin-A1, which plays a role in synaptic vesicle endocytosis and is associated with lipid binding (Huttner and Schmidt, 2000). The *CDH20* gene encodes a type 2 classical cadherin, which is a calcium dependent cell-cell adhesion glycoprotein and a prime candidate for tumor suppression (Kools et al., 2000). A study on childhood obesity in Hispanic children found this gene to have a suggestive association with energy balance (*P*-value = 5.60E^−6^) (Comuzzie et al., 2012). The *AACS* gene (acetoacetyl-CoA synthetase-like gene) mediates activation of ketone bodies for synthesis of fatty acid and cholesterol. *AACS* is potentially being regulated by the leptin signaling pathway via the brain and consequently a cause of metabolic disorders (Narishima et al., 2009). The *ADPGK* gene (ADP-dependent glucokinase-like gene) regulates T-cell activation, affecting the glycolytic metabolism (glucose uptake) (Kamiński et al., 2012). All genome-wide significant GWA study results are presented in Additional file 1.

### Validation of GWAS results by LDLA and haplotype block analyses

**Figure 3:**
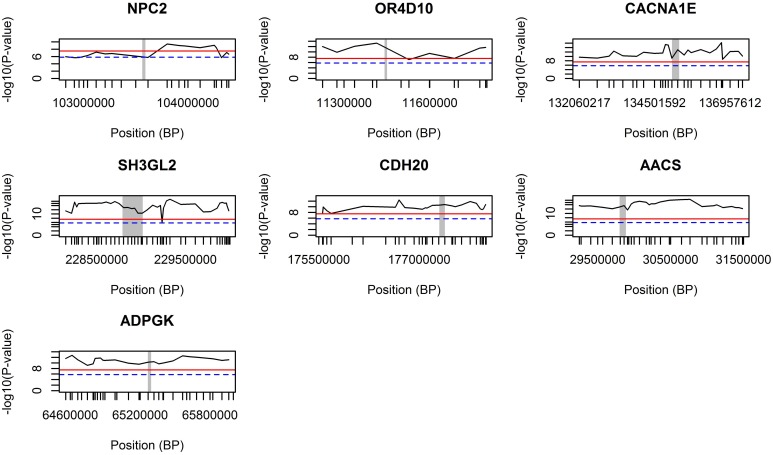
**LDLA regional plots for regions around seven selected genes**. The concerning gene is vertically marked with a gray bar. The blue dash line indicates a suggestive significance threshold with adjusted Bonferroni correction at *P* = 1.58E^−6^ and the red line indicates a highly significant threshold with adjusted Bonferroni correction at *P* = 3.17E^−8^.

The GWA study revealed several highly associated SNPs, in which, among others, seven genes were chosen after their co-location with the highest genome-wide significant SNPs (based on *p*-values; Table 1). The genomic regions around those seven genes were further validated using the combined LDLA. We then interrogated the LDLA validated regions using haplotype block analyses for genetic variants and genes that are in close vicinity and in high linkage disequilibrium. The LDLA approach modeled QTLs in the middle of successive marker pairs located within the above mentioned genetic regions, and results indicated that QTLs modeled in all possible positions within these seven detected genes passed the suggestive significance threshold (*P*-value = 1.6E^−6^) and, except for *NPC2*, passed the highly significant threshold (*P*-value = 3.2E^−8^). Regional plots of these seven indicated genes are presented in Figure 3.

**Figure 4:**
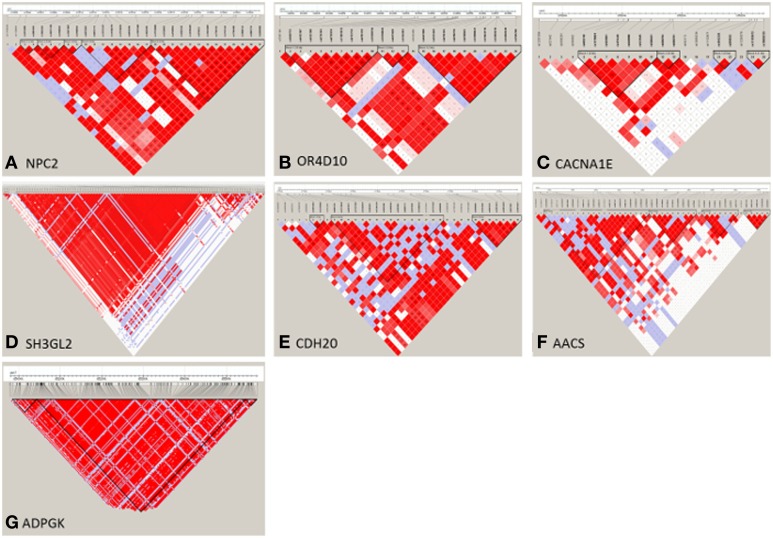
**Haploview representation of regional haplotype block estimates of seven selected regions, detected by GWA analysis**. On the vertical line the SNPs present in the selected region, and the triangles (black lines) represent the haplotype blocks. **(A)** NPC2 gene is located in a 8-SNP haplotype block of 4 Kb size on Chromosome 7; **(B)** OR4D10 gene is located in a 9-SNP haploype block of 10 Kb size on Chromosome 2; **(C)** CACNA1E gene is located in a 7-SNP haplotype block of 8 Kb size on Chromosome 9; **(D)** SH3GL2 gene is located in a 129-SNP haplotype block of 67 Kb size on Chromosome 1; **(E)** CDH20 gene is located in a 16-SNP haplotype block of 46 Kb size on Chromosome 1; **(F)** AACS gene is located in a 9-SNP haplotype block of 26 Kb size on Chromosome 14; **(G)** ADPGK gene is located in a 204-SNP haplotype block of 500 Kb size on Chromosome 7.

After LDLA confirmation of the seven genomic regions detected by GWA analysis, we further examined the validated regions by investigating possible haplotype block structures. The *NPC2, OR4D10*, and *CACNA1E* genes were located in small haplotype blocks. The *NPC2* gene is located on chromosome 7. In the evaluated region (103.566–103.584 Mb) there are five small haplotypes, wherein the *NPC2* gene is located on a small haplotype block of 4 Kb containing eight SNPs (Figure 4A). This haploblock does not contain any other genes. The *OR4D10* gene is located on chromosome 2, and in the evaluated region (11441–11458 Mb) there are three small haplotype blocks, and the *OR4D10* gene is located in a haplotype block of 10 Kb containing nine SNPs (Figure 4B). There are no other genes present in this haplotype block. The *CACNA1E* gene is located on chromosome 9, and in the evaluated region (13596–135224 Mb) there are four small haplotype blocks, where *CACNA1E* is located in a haplotype block of 8 Kb containing seven SNPs (Figure 4C). This haplotype block contains no other genes. The *SH3GL2, CDH20*, and *AACS* genes were located in moderately sized haplotype blocks. The *SH3GL2* gene is located on chromosome 1, and in the evaluated region (228866–228993 Mb) there are eight small haplotype blocks. *SH3GL2* is located in the largest haplotype block (67 Kb) containing 129 SNPs (Figure 4D), containing one more gene: *CNTLN* (centlein, centrosomal protein). The *CDH20* gene is also located on chromosome 1, and in the evaluated region (177320–177418 Mb) there are three small haplotype blocks. The *CDH20* gene is located on a haplotype block of 46 Kb containing 16 SNPs (Figure 4E), but does not contain any other genes. The *AACS* gene is located on chromosome 14, and we detected four blocks in the evaluated region (29775–29874 Mb), where the *AACS* gene was located on the haplotype block of 26 Kb containing nine SNPs (Figure 4F), but no other genes were present in this haplotype block. The *ADPGK* gene was located on chromosome 7 in a large haplotype block of 500 Kb containing 204 SNPs (Figure 4G). In this haplotype block (65060–65560 Mb) there are several other genes located: *BBS4* and *ARIH1* are downstream of *ADPGK, TMEM202, HEXA, PARP6, PKM*, and two uncharacterized proteins are upstream of *ADPGK*. According to the haplotype block analysis we can conclude that we can be reasonably confident about the genes we pinpoint, however, with the *ADPGK* gene we may have a noise because of the many other genes in the same haplotype block. Previous research has shown an average haplotype block size of 400 Kb, of which most between 100 and 400 Kb, for several production pig lines (Veroneze et al., 2013). Since this is an F2 population, we expected even larger haplotype block sizes then detected here.

### Pathway detection

Pathway analysis was performed using genes located within a flanking distance of 20 Kb of the genome-wide significant SNPs (*P*-value = 2.44E^−8^) identified via GWA analysis. Initially, analysis using the NCBI2R R-package only identified *metabolic pathways* (*P*-value = 0.08) that was not significant after Bonferroni correction was applied to account for multiple comparisons. Subsequently, GeneNetwork (http://www.genenetwork.nl) was used to detect overrepresented pathways; and phenotypes and tissues associated with these based on publicly available expression data. GeneNetwork analyses identified various cellular, transport related processes, as e.g., the GO biological processes *cell chemotaxis* (*P*-value = 6E^−6^), and the KEGG pathway *endocytosis* (*P*-value = 1E^−6^). The associated phenotypes [using data from the Mouse Genome Informatics (MGI) database] included inflammatory related phenotypes, e.g., *decreased inflammatory response* (*P*-value = 3E^−5^), *demyelination* (*P*-value = 3E^−5^), *decreased macrophage cell number* (*P*-value = 4E^−5^), and *decreased tumor necrosis factor secretion* (*P*-value = 8E^−5^). Furthermore, the tissue expression database shows a strong role for the nervous system, as the *spinal nerve roots* are the most significant associated tissue (*P*-value = 9E^−7^). Finally, we used GOEAST to investigate gene ontology (GO) terms, in which the genome-wide significant genes were overrepresented. Results from these analyses indicate significant enrichment for GO terms associated with the glucose/insulin metabolism in the Biological Processes category, e.g., *negative regulation of insulin secretion* (*P*-value = 2.39E^−7^, log odds = 4.46) and *cellular response to glucose stimulus* (*P*-value = 2.94E-11, log odds = 4.50). Other overrepresented GO terms in the Biological Processes category were *glycolysis* (*P*-value = 6.01E^−7^, log odds = 3.33), and *skeletal muscle fiber development* (*P*-value = 1.91E^−5^, log odds = 3.65). In the Cellular Component category, we did not find highly significant GO terms. In the Molecular Function category the *calcium-dependent protein serine/threonine phosphatase activity* (*P*-value = 2.42E^−12^, log odds = 6.44), *pyruvate kinase activity* (*P*-value = 1.62E^−13^, log odds = 6.32), and *ferric iron binding* (*P*-value = 5.89E^−8^, log odds = 4.69) were most significant.

### Network analysis using the wish network method

GWA studies have several limitations, as discussed above: many SNPs are eliminated as they do not reach the strict genome-wide significance thresholds and they do not take genetic networks based on gene–gene interactions into account. We therefore used the WISH network method to identify clusters of highly interconnected SNPs (modules), using the Duroc ^*^ Göttingen Minipig (DM) intercross population. Detected modules were further investigated using various pathway detection approaches, to indicate their biological relevance.

#### Network construction

**Figure 5:**
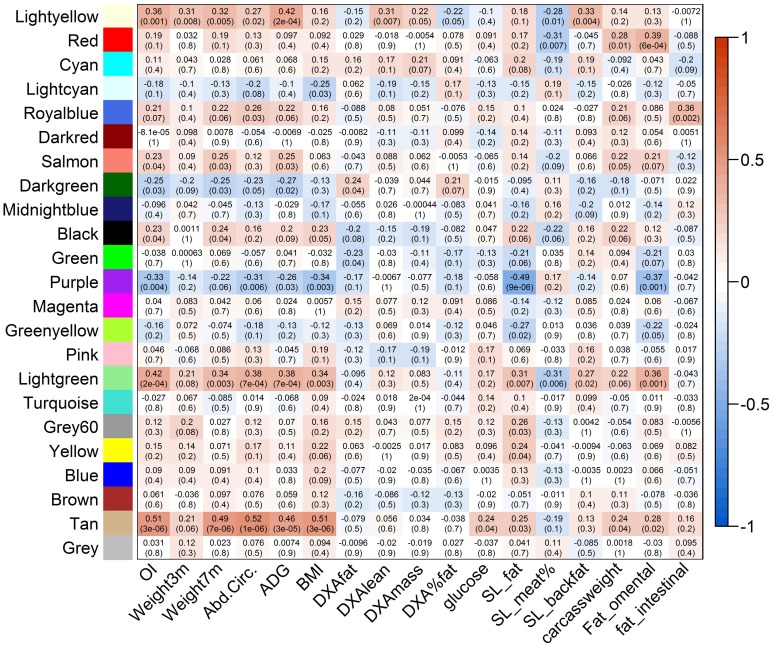
**Genome-wide Association Matrix (GMAT) of modules detected using the WISH network method**. On the y-axis the detected modules are visualized, and on the x-axis the different phenotypes (OI and breeding values of 16 obesity-related phenotypes) are presented. In the matrix the Module-Trait Relations (MTRs) are presented with the subsequent *p*-values. MTRs are colored red in case of a strong positive correlation between the Module and the Trait, and colored blue in case of a strong negative correlation between the Module and the Trait.

Data reduction was based on the genome-wide significance (*P*-value < 0.05) and the connectivity of the SNPs, resulting in a selection of 2500 SNPs for network construction. Using the WISH network method based on genotype correlations, we detected 17 modules of at least 50 SNPs per module. The module eigenSNP was calculated based on the first principal component, which explained 48–78% of the variation in the modules. We then selected biologically interesting modules based on the Module-Trait Relationship (MTR), which was calculated as the correlation between the module eigenSNP and the traits of interest: the OI and 16 other obesity-related traits. Six modules were selected for downstream analysis based on the Genome-wide Module Association Matrix (GMAT), as they had a significant correlation with the OI, and a correlation of >0.4 with at least one other obesity-related trait (Figure 5). Selected modules and their MTR with OI were: Tan module (MTR_OI_ = 0.51), Lightgreen module (MTR_OI_ = 0.42), Lightyellow module (MTR_OI_ = 0.35), Purple module (MTR_OI_ = −0.33), the Royalblue module (MTR_OI_ = 0.21), and the Red module (MTR_OI_ = 0.19).

#### Downstream analysis of detected modules

Genes located within 20 kb of SNP present in selected modules were detected using the R-package NCBI2R. We performed pathway analysis using NCBI2R, GOEAST and GeneNetwork for all six selected modules resulting from WISH network construction.

The Tan module included 84 SNPs, corresponding to 64 genes. GOEAST analyses resulted in the identification of several significant GO terms in the actin filament pathway (e.g., *actin crosslink formation, P*-value = 2.59E^−8^) which is not directly associated with obesity. GeneNetwork analyses identified some overrepresented phenotypes related to diabetes (i.e., *increased susceptibility to autoimmune diabetes, P*-value = 4.13E^−4^; and *abnormal pancreatic beta cell morphology, P*-value = 6.58E^−4^), which may be resulting from the overrepresented Biological Process GO term *branched chain family amino acid metabolic process* (*P*-value = 6.98E^−5^), as those amino acids are associated with the metabolic homeostasis (Wang and Guo, 2013).

The Lightgreen module consists of 77 SNPs representing 47 genes. The Molecular Function category in GOEAST shows one highly overrepresented GO term: *purinergic receptor activity* (*P*-value = 7.21E^−25^). Purinergic receptors have been implicated in several different functions as, e.g., learning and memory, locomotor and feeding behavior, and sleep. Two genes present in this pathway are *P2RX7* and *ADORA2A*. *P2RX7* is encoding the protein P2X purinoreceptor 7, which have been implicated in, e.g., neuronal cell death and inflammation (Skaper et al., 2010). Moreover, the *P2X7* gene has been associated with diabetes, e.g., by its influence on the regulation of beta cells (Glas et al., 2009). The *ADORA2A* (adenosine A_2A_ receptor) gene is coding a protein which is a receptor subtype for adenosine. It regulates blood flow to the myocardium by vasodilation of the coronary arteries, potentially leading to hypotension. Besides, it has a neuronal effect through its expression in the brain, associated with, e.g., anxiety and depression (Ledent et al., 1997). A direct link with obesity or type 2 diabetes has not been shown, to our knowledge. Furthermore, the Biological Process category in GOEAST also shows the overrepresented GO term *fructose 2,6-bisphosphate metabolic process* (*P*-value = 3.95E^−20^), possibly because of the presence of the PFKFB4 gene. PFKFB4 regulates the concentration of fructose 2,6-bisphosphate, which plays a key role in glycolysis (Uyeda et al., 1982).

The Lightyellow module consists of 72 SNPs representing 45 genes. The GOEAST analysis only reveals some slightly significant overrepresented GO-terms, as, for example, the Molecular Function *alpha-glucosidase activity* (*P*-value = 2.91E^−7^). The *GANC* gene is present in this module and GO-term, encoding the neutral alpha-glucosidase C enzyme. This enzyme plays a major role in the glycogen metabolism and because of its effect on the absorption of sugars from the gut, alpha-glucosidase inhibitors are used in the treatment of type 2 diabetes (van de Laar, 2008).

The Purple module is the largest module, consisting of 104 SNPs and representing 84 genes. The NCBI2R pathway analysis detected one, more general, pathway: *metabolic pathways* (*P*-value = 0.06, not significant after multiple-testing correction). Using the GOEAST analysis, several GO-terms were detected as overrepresented, e.g., *microtubule anchoring* (*P*-value = 4.61E^−31^) and *gamma-tubulin binding* (*P*-value = 1.81E^−26^).

The Royalblue module is the smallest module, consisting of 57 SNPs representing 49 genes. The NCBI2R pathway analysis came up with many significant overrepresented pathways, which were also significant after multiple-testing correction. Most significant were immune-related pathways, e.g., *antigen processing and presentation* (*P*-value = 7.6E^−6^) and *herpes simplex infection* (*P*-value = 1.85E^−5^), while others were more directly related to the OOR diseases, e.g., *type I diabetes mellitus* (*P*-value = 5.8E^−4^). Using GOEAST several GO terms in the Biological Processes category were detected as overrepresented, e.g., *collagen-activated signaling pathway* (*P*-value = 2.03E^−72^) and *smooth muscle migration* (*P*-value = 4.06E^−66^).

Lastly, the Red module is consisting of 82 SNPs representing 54 genes. GOEAST detected some overrepresented GO-terms associated with the monosialoganglioside sialytransferase activity (*P*-value = 5.22E^−12^). The *ST3GAL4* gene, present in this module, has been associated with this GO-term. This gene has been associated to glycoprotein biosynthesis and cell surface glycobiology, which may be associated to the concentration of liver enzymes which is often disturbed by diseases like obesity and give an increased risk for, e.g., type 2 diabetes (Chambers et al., 2011).

#### Differentially wired (DW) networks of SNPs

Differentially wired (DW) or connected genes are genes which are highly interconnected with other genes in one extreme subgroup (hub genes), while lowly interconnected with other genes in the other extreme subgroup. Here, we examined the differential connectivity (k_diff) of SNPs between the lean subgroup and the obese subgroup. These DW genes in one group vs. the other would be biologically interesting candidates as they might point to differences in underlying genetic regulation for manifestation of the obesity or leanness. In total, 55 SNPs showed an absolute differential connectivity above 0.6, which resulted in the detection of 36 genes (including 10 uncharacterized proteins) within a flanking distance of 20 Kb. We here present the genes strongly associated with obesity or obesity-related diseases before, all DW SNPs and corresponding genes are presented in Additional File 2.

Several DW genes were hub genes in the lean sub network, but had a low interconnectivity in the obese sub network, e.g., *UBR1, PNPLA8*, and *CTNAP2*. The *UBR1* gene (k_diff = 0.62) encodes a member of the E3 ubiquitin ligase family, which functions in the N-end rule pathway. This pathway has been associated with various functions, e.g., the control of apoptosis (Ditzel et al., 2003). Moreover, Ubr1 knockout mice also demonstrate subtle effects in muscle protein degradation and fat metabolism (Kwon et al., 2001). The *PNPLA8* gene (k_diff = 0.62) encodes a member of the patatin-like phospholipase domain containing proteins. These phospholipases catalyze the cleavage of fatty acids from membrane phospholipids. The *CNTNAP2* gene (k_diff = 0.62) encodes contactin-associated protein-like 2, a protein which functions in the nervous system as cell adhesion molecules and receptor. A neuronal impact on obesity is suggested by this gene, by the influence on the potassium channel at the nodes of Ranvier, resulting in the regulation of diet-induced obesity (Buchner et al., 2012).

On the other hand, DW genes that were detected with a low interconnectivity in the lean sub network were in fact hub genes in the obese sub network. Two of these DW genes have previously been reported to be associated with obesity and diabetes, respectively: the *NUCB2* gene and the *BCL11A* gene. The nucleobindin-2 (*NUCB2*) gene (k_diff = −0.80) is a precursor of nestafin-1, a hypothalamic anorectic neuropeptide. It has recently been discovered that this neuropeptide is expressed in, e.g., pancreatic islet cells and the central nervous system. It seems to play an important role in hypothalamic pathways regulating food intake and energy homeostasis, and it has been shown to play an important role in regulation of food intake in obese individuals (Abaci et al., 2013). Moreover, it has been shown that nesfatin-1 is expressed in the same human gastric X/A-like cells as ghrelin, a hunger-stimulating hormone. The expression of nestafin-1 and ghrelin is differentially regulated under obese conditions: nestafin-1 increases and ghrelin decreases with an increasing BMI, toward a further adaptive change that may counteract further body weight increase (Stengel et al., 2013). The *BCL11A* gene (k_diff = −1.00) encodes the B-cell lymphoma/leukemia 11A protein. The corresponding mouse gene has been associated with leukemia, through its interaction with *BCL6*. However, this gene has also been detected in GWA studies to be associated with type 2 diabetes and pancreatic β-cell function (Simonis-Bik et al., 2010; Langberg et al., 2012).

## Discussion

In this systems genetics analysis using an F2 porcine model of OOR diseases, we used several methods to identify underlying genetic variants, modular networks, biologically relevant pathways, and hub genes. All these analyses were based on the OI, an aggregate genetic value constructed by combining the estimated breeding values of nine different obesity-related phenotypes, thought to be highly relevant in defining both overall and visceral obesity. We used the principles of genetic selection index method often used in animal breeding to construct an aggregate genetic index for obesity in porcine model. To our knowledge, this is the first study to develop such an aggregate (genetic) OI for systems- and network genetics investigations. The porcine model was subject to extensive phenotyping, which is either not possible or expensive in human populations. Previous investigations have shown that a large proportion of variation in these phenotypes is genetically determined (Kogelman et al., 2013), thereby offering an opportunity to exploit this resource to provide novel insights into the genetic complexity of the disease. By combining a key number of the obesity phenotypes into one, we were able to investigate this complex disease with one simplified aggregate phenotype. Moreover, the OI allows selection of extremely obese and extremely lean animals, resulting in a greater power to detect genes influencing obesity.

### GWA study and validation of identified regions

A single SNP association approach was implemented via GWA analysis on the OI, and subsequently seven selected GWAS regions were validated using combined LDLA, and further investigated by analyzing haplotype blocks in these regions using Haploview. A large number of SNPs (representing 289 genes) were found to be significantly associated with OI via GWA. The high number of genome-wide significant SNPs is likely due to the use of the OI that combines nine different phenotypes. Consequently, we do expect to find a greater number of SNPs for this aggregate phenotype than for a situation where only one phenotype is analyzed. Moreover, since OI represents only the polygenetic (or estimated breeding) value for each phenotype used in its construction, it is likely that re-regressing on a regressed “phenotype” will yield inflated estimates of the regression coefficient. This explains the high number of significant SNPs in the GWA study, but also the very small *p*-values obtained via the LDLA approach. However, since regression was used only to rank SNPs for data reduction, and regression coefficients were not used for network construction, this did not influence the network analysis.

The resource population was constructed by crossing genetically divergent breeds differing with respect to obesity related phenotypes, followed by sibling mating. The resultant F2 porcine intercross population allows Linkage Disequilibrium (LD) to exploit family information to validate GWA findings. Traditional GWA studies rely solely on across population LD that only extends to short genetic intervals. Consequently, causal variants are hard to identify if they are not located in the immediate vicinity of the markers assayed in a study. Since within family LD extends to larger genetic intervals, analytical approaches that use this information along with across family LD (e.g., combined linkage disequilibrium linkage analyses; LDLA) provides greater power and finer resolution of the candidate loci particularly in genomic regions with low population wide LD (Meuwissen et al., 2002). LDLA is a fine mapping approach that combines both within-family linkage and population-wide LD in one analysis. Since single marker GWA analysis is based on population wide LD and linkage information was available given the 3-generation F2 intercross pedigree, it was a reasonable further step to capitalize on this F2 pedigree structure to validate findings of highly significant regions in the GWA analysis.

Obesity is a very complex trait, with many different involved biological pathways, and consequently a huge number of genes will be related. Many SNPs showed a significant association with OI, and using a flanking distance of 20 Kb, we consequently found many genes located in or nearby those SNPs. As discussed, we would expect that SNPs over a rather long distance will be in LD, as a consequence of using parental lines genetically divergent for two different characteristics (leanness vs. obese) each line having larger LD blocks passed onto F2 pigs. This would mean using a larger flanking distance, consequently detecting even more genes in the regions of associated SNPs. However, we have seen in this study that there are rather small haplotype block sizes, and because of the high number of SNPs detected, we limited gene detection to a flanking distance of 20 Kb.

As expected, several of our GWA study finding could be directly or indirectly linked to obesity. The number of associated genes is also in agreement with other studies: GWA studies alone have indicated at least 37 genes related with BMI, 14 genes with WHR adjusted for BMI, 3 genes with fat percentage, etc. (Fall and Ingelsson, 2014). Only one of our GWA study findings overlap with previous findings, published in Fall and Ingelsson (2014), as for example the *MAP2K5* gene (*P*-value = 2.32E^−9^) on chromosome 1 of the pig genome. This gene has previously been detected by GWA studies on BMI by Speliotes (Speliotes et al., 2010) and Wen (Wen et al., 2012). However, many loci previously reported to be associated with obesity were not detected in our study, e.g., *FTO* and *MC4R* gene. SNPs located in or near both these genes did not survive data quality control. Moreover, many SNPs could not be annotated, mainly due to the limited annotation of the pig genome, which is also a main limitation in the pathway detection among SNPs detected using the GWA study. Pathway analysis did show some biologically relevant pathways, e.g., *negative regulation of insulin secretion*, but unfortunately, the findings of the different pathway methods did not overlap completely. The inadequate knowledge about the annotation of the pig genome also results in limited knowledge about biological pathways present on the pig genome. We therefore chose to identify the genes in close proximity of SNPs, and use those genes in a human pathway setting, by using the human reference genome and annotation information. Furthermore, the variation in results between the different pathway methods is also due to the different reference datasets, as for example GOEAST works with GO terms only, and the NCBI2R method was based on KEGG pathways.

### Network approach

We used the WISH network method to cluster SNPs based on the correlation patterns of genotypes. This systems genetics approach gave us the opportunity to analyze the interactions between thousands of SNPs, and thereby overcome major limitations of GWA studies. GWA studies use a very stringent genome-wide significance threshold, excluding potentially biologically relevant associated SNPs with very small effect sizes. Here, we selected SNPs based on a less stringent nominal *P*-value threshold (0.05), increasing the probability of including SNPs with smaller effect sizes that are biologically relevant for the trait of interest. Moreover, we are not only interested in the detection of single genetic variants associated with obesity, but also in the detection of molecular pathways through which the genetic variants exerts their effects. Because of the exclusion of SNPs with interaction effects on phenotypes in GWA study, the elucidation of important pathways is limited. By using a WISH network method, we are able to include and analyze genome-wide interactions between SNPs and relate them to molecular and cellular functions.

WISH network construction was based on genotypic correlation and since genotypic correlations largely represent LD, part of the SNPs in the network modules were found to be in close physical proximity. However, many SNPs in the modules were not physically co-located. For example, the Tan module has the highest correlation with OI and consists of 84 SNPs. Of these, 9 SNPs could not be mapped to the porcine genome and the remaining 75 SNPs were distributed over all porcine chromosomes, except chromosome 15. As shown in Table 2, there is a fairly equal distribution of SNPs included in this module over different porcine chromosomes. Moreover, in cases where many SNPs are co-located on one chromosome, they cover a large area of the chromosome. This indicates that the WISH network construction based on genotypic correlations does not only capture SNPs physically co-located. However, previous studies have also shown that very distant loci located on different chromosomes can be in LD with each other (Flint-Garcia et al., 2003). Studies have advocated investigating LD between loci, as a SNP highly associated with the disease could be in LD with a causal SNP (Weiss and Clark, 2002). By detecting clusters of SNPs that are in LD with each other, and determining their functional annotation, we attempt to investigate the biological relevance of SNPs in genome-wide LD. The WISH network method may also be applied using an epistatic interaction model, using the regression coefficients of the SNP^*^_i_SNP_j_ interaction term. However, as discussed previously, regression models using a regressed phenotype like OI will likely yield inflated coefficients. Therefore, we did not apply the WISH network method based on epistatic interactions in this study.

**Table 2:** **Distribution of SNPs over the chromosomes in the Tan Module using the WISH network method based on genotypic correlations**.

**Chromosome**	**# SNPs**	**Location**	
		**Minimum**	**Maximum**
1	17	87.621.858	291.903.522
2	11	2.338.853	145.102.337
3	3	74.111.456	141.121.427
4	7	21.818.546	142.317.862
5	3	4.874.980	10.686.539
6	4	43.090.860	126.991.726
7	4	87.893.776	97.500.922
8	2	20.144.538	109.213.653
9	4	48.813.606	132.656.946
10	1	58.238.109	58.238.109
11	4	37.160.369	82.659.616
12	2	52.678.657	54.767.975
13	5	31.119.717	172.601.927
14	2	62.514.781	63.414.448
16	3	8.438.953	68.583.123
17	1	9.342.342	9.342.342
X	1	6.909.025	6.909.025

In total six WISH network modules were further examined using different pathway analysis methods. Those methods show different results which could be directly or indirectly related to obesity and obesity related diseases. As expected, many pathways were related to metabolic processes (e.g., *fructose 2,6-bisphosphate metabolic process* and *branched chain family amino acid metabolic process*). The variety of results show the complexity of the disease under study, as many different metabolic associated pathways and GO terms show up in the different modules. To further investigate the relation with the insulin pathway, and shown the additional value of systems genetics approaches, we looked further into the connectivity's of the SNPs present in the Lightgreen Module, as this one showed associations with, e.g., the glycolysis pathway. Three out of the seven highest connected SNPs were located in the *FRMD4B, ADAMTS9*, and *XCR1* genes. Those genes were all previously related to the insulin pathway or diabetes, where *XCR1* (encoding a chemokine receptor) even linked obesity to insulin resistance before (Ota, 2013). The *FRMD4B* gene is part of the GRP1 signaling complex, thereby recruited in response to insulin receptor signaling, but genetic variants have also been associated with heart failure (Matkovich et al., 2010). The *ADAMTS9* gene has been associated to Type 2 Diabetes in a meta-analysis of genome-wide association data (Zeggini et al., 2008). Those genes show in the network construction a perfect correlation (of 1) with each other, and as they lie on the porcine genome in a region of approximately 18 Mb of each other, it is likely that they are within the same haplotype block. However, the integrative systems genetics approaches also shows the potential of further investigating those genes, and gaining knowledge about the genetic architecture of complex traits. We examined the differential wiring of SNPs between two subgroups that are genetically lean and obese. These DW genes in one group vs. the other would be biologically interesting candidates as they might point to differences in underlying genetic regulation for manifestation of the obesity or leanness. We detected 55 DW SNPs and 36 co-located genes within 20 kb (e.g., *UBR1, PNPLA8*, and *CTNAP2*), which have significant implications for development of obesity and or obesity-related diseases, as shown in additional file 2. The WISH network was able to identify GO terms and pathways which were not identified by pathway analysis of the GWA study results, and moreover, none of the DW genes were identified using the GWA study.

In general, the study describes several obesity related genes and pathways that accord with the complexity of the disease. As this F2 pig resource population is extensively phenotyped, further studies could yield novel biological insights underlying the association between phenotypes used in this study and the identified genes. The results of this study overlap with previous human findings, while also identifying novel genes not previously known to be associated with human obesity. However, further validation of those loci will be needed to confirm the association with obesity, and their exact function in biological pathways. Once validated, these findings could potentially be extended to humans in order to improve the treatment of obesity and eventually reduce complementary problems resulting from obesity.

## Conclusion

This is the first study to develop an aggregate (genetic) OI based on the principles of quantitative genetics and animal breeding, to study genetic of obesity. We demonstrated the potential of network-based systems genetics approaches to reveal biological and genetic background of complex phenotypes that is otherwise not identified via traditional genetics/genomic analyses. Here we performed GWA analysis, validated these results via LDLA and evaluation of the haplotype block sizes, and subsequently performed enrichment analysis to identify biological pathways associated with obesity and obesity related traits. Subsequently, scale-free WISH networks were constructed and different clusters of highly interconnected SNPs that were putatively related to obesity and related diseases were identified, demonstrating the importance of genetic interactions in obesity. We have also examined the differential connectivity or wiring of SNPs between two subgroups that are genetically obese and lean and detected 36 co-located genes, which have significant implications for development of obesity and or obesity-related diseases. Many genes with diverse functions and consequently many different pathways were identified by WISH and differential wiring approaches that were not detected by traditional GWA analyses, thereby demonstrating that integrative systems approaches could potentially yield novel insights into the genetic determination of obesity and its relation to other diseases. This study, to the best of our knowledge, is the first network-based systems genetics analyses on an experimental pig population in which a wide range of obesity traits have been investigated, and reaffirms the complexity associated with obesity as a disease.

## Materials and methods

**Figure 6:**
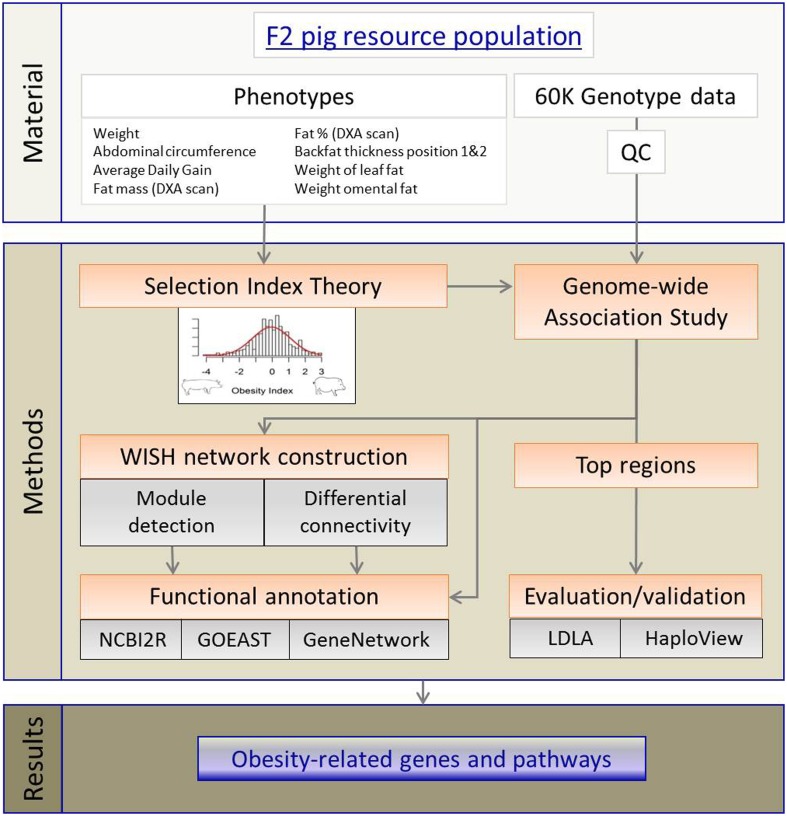
**Workflow visualizing the different methods to detect obesity-related genes and pathways**.

The complete workflow, from materials to results, is presented in Figure 6. The core materials for this study are the purpose-built F2 pig model for obesity, high-throughput genotypic data, and nine selected obesity phenotypes. Using these materials, we developed first an aggregate (genetic) OI and applied GWAS to the OI. The outputs of GWAS were then used in WISH network construction. The LDLA methods were used validate top genomic regions before conducting pathway, network and functional annotation analyses. These approaches then resulted in identification of novel obesity-related genes and pathways which provide deeper understanding of genetic control of obesity development.

### The F2 pig resource population: phenotype and genotyping

An F2 pig resource population, genetically divergent for OOR traits, was established as described previously (Kogelman et al., 2013). Briefly, the F2 pig resource population was created using Danish production pig breeds, i.e., purebred Yorkshire (YY) and Duroc (DD) sows from a DanBred breeding herd and Göttingen Minipig (MM) boars from Ellegaard A/S in the parental generation. The production pigs have been selected for leanness and growth among other traits during the last 60 years, whereas Göttingen Minipigs are bred principally for their small size and ease of handling and are prone to obesity. They are also known to share the metabolic impairments seen in obese humans (Wang and Guo, 2013). The F2 pig resource population consists of 24 parental animals, 78 F1- and 454 F2-animals. The resulting population of 556 animals can be divided into Duroc^*^Göttingen Minipig (DM, 279 pigs) and Yorkshire^*^Göttingen Minipig (YM, 277 pigs) subpopulations. Animal care and maintenance was conducted according to the Danish “Animal Maintenance Act” (Act 432 dated 09/06/2004). This F2 pig resource was extensively phenotyped for several OOR traits that included weight, conformation, dual energy x-ray absorptiometry (DXA) scanning and slaughter measurements. Genetic parameters estimated via this F2 pig resource population revealed normally distributed genetic values within those OOR traits, and the potential for further genomic and system genetic investigations (Kogelman et al., 2013). Descriptive statistics and heritabilities of the main traits of interest in this study are presented in Table 3.

**Table 3:** **Descriptive statistics and heritabilities of main fatness-related traits in the pig resource population, as published in Kogelman et al. (2013)**.

**Trait**	**Unit**	**Abbreviation**	***N***	**Mean**	***SD***	***h*^2^**
**WEIGHT**						
2 months	Kg	WT_2m_	439	12.45	4.52	0.78
7 months	Kg	WT_7m_	405	94.45	17.51	0.39
**Average daily gain**	Kg/day	ADG	403	0.44	0.08	0.54
**Abdominal circumference**	Cm	ABD_7m_	404	122.7	10.56	0.28
**BMI^a^**	Kg/cm^2^	BMI_7m_	403	132.86	20.71	0.23
**DXA**						
Fat	Kg	DXA_fat_	438	22.92	8.53	0.43
Lean	Kg	DXA_lean_	438	100.42	37.33	0.71
Mass	Kg	DXA_total_	438	123.34	44.93	0.67
% fat	%	DXA_%fat_	438	18.64	2.87	0.57
**Fasting glucose**	Mmol/L	FGL	146	4.50	2.28	0.49
**SLAUGHTER**						
Carcass weight	Kg	SL_cw_	358	56.33	11.77	0.54
Meat percentage	%	SL_%meat_	330	43.39	6.65	0.18
Weight leaf fat	Kg	SL_fat_	396	2.59	1.10	0.23
Backfat 1^b^	Mm	SL_bf1_	330	31.90	8.54	0.22
Backfat 2^c^	Mm	SL_bf2_	330	35.44	9.12	0.23
Omental fat	Kg	SL_fat_om_	257	352.72	145.28	0.52
Intestinal fat	Kg	SL_fat_int_	219	19.88	8.32	0.08

a BMI, body mass index, calculated as [weight/(length)^2^].

b Backfat measured between third and fourth lumbar vertebra, 8 cm off midline.

c Backfat measured between third and fourth last rib, 8 cm off midline.

Blood was collected from all pigs in the F2 pig resource population from the jugular vein. Genomic DNA was extracted from EDTA stabilized blood using a simple salting out procedure (Miller et al., 1988). Genotyping was performed by GenoSkan A/S, Tjele, Denmark using the Illumina 60K porcine SNP-chip. Quality control (QC) was performed in the R-package GenABEL (Aulchenko et al., 2007), resulting in the exclusion of seven animals due to low call rate (≤0.05), and seven animals due to too high identical by state (IBS ≥ 0.95). Furthermore, 3240 markers were excluded because of a low call rate (≥0.05), 7615 markers were excluded because of their low minor allele frequency (MAF ≤ 0.05), and 4723 markers were excluded as they were not in Hardy–Weinberg equilibrium (*P*-value < 1E^−5^). This resulted in the analysis of 40,910 markers and 538 animals.

### Obesity index based on breeding values

To maximize genetic progress, selection indices are extensively used in animal breeding (Cameron, 1997) to select animals with desirable genotypes for a particular phenotype of interest. The selection index theory was used to create one genetic value representing the degree of obesity for all animals, resulting in one more distinct phenotype for obesity. Using the estimated variance components, combining several OOR traits into one aggregate genetic value for all animals in the F2 pig resource population, we created the OI. Traits used for construction of the OI were: weight at slaughter age (WT_7m_), abdominal circumference at slaughter age (ABD_7m_), average daily gain (ADG), estimated fat mass at DXA (DXA_fat_), estimated percentage of fat at DXA scanning (DXA_%fat_), backfat thickness at position 1 (SL_bf1_) and position 2 (SL_bf2_), weight of leaf fat at slaughtering (SL_fat_), and omental fat at slaughtering (SL_fat_om_). These phenotypes were selected to collectively represent and be associated with “obesity” as an excessive amount of adipose tissue.

By combining the estimated breeding or genetic values (EBVs) for several traits per animal, and weighting individual genetic values of each traits by their relative biological weight (*v*), one aggregate genetic value per animal is calculated representing all phenotypes as:

$$I=b_{1}x_{1}+b_{2}x_{2}+\cdots +b_{j}x_{j}=b^{\prime }x$$

Where *I* is the selection index, *b_j_* is the selection index coefficient (weight) for the *j*th observation, and *x_j_* is the *j*th phenotypic observation. The selection index coefficient *b* is calculated based on the phenotypic and genetic (co-)variance components, multiplied by a biological assigned weight for the particular traits:

$$b=P^{-1}Gv$$

Where **P^−1^** is the inverse matrix of the phenotypic (co-)variances, **G** is the matrix of the genotypic (co-)variances and **v** is a vector with the biological assigned weights. The **P** and **G** matrices are constructed as follows:

$$P=\left[\begin{array}{cccc} \sigma_{P1}^{2} & \sigma_{P1,2} & \ldots & \sigma_{P1,9}\\ \sigma_{P2,1} & \sigma_{P2}^{2} & \ldots & \sigma_{P2,9}\\ \ldots & \ldots & \ldots & \ldots \\ \sigma_{P9,1} & \sigma_{P9,2} & \ldots & \sigma_{P9}^{2} \end{array}\right],\;G=\left[\begin{array}{cccc} \sigma_{G1}^{2} & \sigma_{G1,2} & \ldots & \sigma_{G1,9}\\ \sigma_{G2,1} & \sigma_{G2}^{2} & \ldots & \sigma_{G2,9}\\ \ldots & \ldots & \ldots & \ldots \\ \sigma_{G9,1} & \sigma_{G9,2} & \ldots & \sigma_{G9}^{2} \end{array}\right]$$

where σ^2^_*Pi*_ is the phenotypic variance of the *i*th trait, σ_*Pi,j*_ is the phenotypic covariance between the *i*th and *j*th trait, σ^2^_*Gi*_ is the genotypic variance of the *i*th trait, and σ_*Gi,j*_ is the genotypic covariance between the *i*th and *j*th trait. The phenotypic and genetic (co-)variance components were estimated using a series of bivariate animal models for all combinations between selected traits for the OI, as presented in our previous study (Kogelman et al., 2013); all models were implemented using ASReml (Gilmour et al., 2009).

As described earlier, each trait was assigned a biological weight (v) based on biological assumptions that was used to calculate the selection coefficient: weight at 7 months of age (v = 0.1), abdominal circumference at 7 months of age (v = 0.1), average daily gain (v = 0.1), body fat estimated by DXA scanning (v = 0.5), percentage of body fat estimated by DXA scanning (v = 0.5), weight of leaf fat at slaughter (v = 0.8), back fat thickness at position 1 (v = 1), back fat thickness at position 2 (v = 1), and weight of omental fat at slaughter (v = 0.8).

Accordingly, OI is defined as:

$$\begin{array}{l} \text{OI}={\text{b}}_{\text{WT7m}}*X_{\text{WT7m}}+{\text{b}}_{\text{ABD7m}}*X_{\text{ABD7m}}+{\text{b}}_{\text{ADG}}*X_{\text{ADG}}\\ \;+{\text{b}}_{\text{DXAfat}}*X_{\text{DXAfat}}+{\text{b}}_{\text{DXA}\%\text{fat}}*X_{\text{DXA}\%\text{fat}}+{\text{b}}_{\text{BF1}}*X_{\text{BF1}}\\ \;+{\text{b}}_{\text{BF2}}*X_{\text{BF2}}+{\text{b}}_{\text{SLfat}}*X_{\text{SLfat}}+{\text{b}}_{\text{SLfat}\_\text{om}}*X_{\text{SLfat}\_\text{om}} \end{array}$$

where OI is the Obesity Index, b is the selection index coefficient calculated using the (co-)variance components and biological weight (v), and **X** is the estimated breeding value of the selected traits. The estimated breeding values, representing the animals deviation from the mean of the population, were estimated using the variance component estimation models presented in Kogelman et al. (2013) using ASReml (Gilmour et al., 2009).

### Genome-wide association analysis

The R package GenABEL (Aulchenko et al., 2007) was used to test the association between individual SNPs distributed throughout the porcine genome, and the OI using the complete F2 pig resource population. Since the OI is derived from estimated genetic (breeding) values calculated after correcting for population structure and all other environmental and fixed effects (see Kogelman et al., 2013) a simple regression of the OI on SNP genotypes was used to perform GWA analysis to avoid double- or over-correction. The basic linear model was:

y=μ+g+e

where *y* = OI, μ = the phenotypic mean, *g* = the SNP genotypes (coded as 1 and 2, 0 for missing), and *e* = the model errors. We calculated the Bonferroni corrected *p*-values by dividing the resulting *p*-value by the total number of SNPs passing QC thresholds (as described above), resulting in a suggestive association at *P*_adj_ = 1.22E^−6^ (0.05/number of SNPs) and a highly significant *p*-value at *P*_adj_ = 2.44E^−8^ (0.001/number of SNPs).

### Validation of GWA study findings by LDLA and haplotype block analyses

The genomic regions with most highly genome-wide significance identified by GWA study (*P*_adj_ = 2.44E^−8^) were further validated using the combined LDLA approach (Meuwissen et al., 2002). Regions were selected when they were in the top 10 GWA results (most highly significant) and the associated gene had a biological role (in-) directly associated to obesity. Length of the regions was arbitrary taken, in such a way that several SNPs up- and downstream were investigated. Statistical significance using the LDLA approach was calculated via a likelihood ratio test of the full model (OI regressed over the phenotypic mean and Identity by Descent probabilities of chromosomal segments flanked by successive marker pairs) vs. the null model (containing only the phenotypic mean). The Identity by descent probabilities were estimated using a linkage disequilibrium multilocus iterative peeling (LDMIP) algorithm described in Meuwissen and Goddard (2010). Furthermore, we identified haplotype blocks in the genomic regions that were detected as being highly significant by both GWA and LDLA analyses, as they are likely to be the most promising genomic regions harboring genetic variants affecting OI. The structure of haplotype blocks in this F2 pig resource population was determined and plotted using Haploview software (v4.2) (Barrett et al., 2005). Pairs were defined as being in “strong LD” using an upper confidence bound on D′ > 0.98 and a lower confidence bound on D′ < 0.7.

### Wish network construction and analysis

We previously published the WISH network method based using whole genome genotype data, giving the opportunity to identify clusters of highly interconnected SNPs (modules) and relate them to phenotypes (Kogelman and Kadarmideen, 2014). We applied these methods to construct WISH networks based on genotypic correlations, using the pipeline as presented previously, in order to identify biologically relevant pathways underlying obesity and obesity related traits in a subpopulation of the pig resource population. The QC of the high-throughput genotype data was performed with the same parameters as for GWA study, resulting in 40,194 SNPs and 266 pigs from the Duroc^*^Göttingen Minipig intercross.

For network construction all SNPs with a genome-wide significance below 0.05 were selected for network construction, resulting in the selection of 9485 SNPs. Animals were selected based on their OI: 75 animals were selected based on an extreme OI (25 low OI, 25 intermediate OI, and 25 high OI). These selections resulted in a 75 * 9485 matrix of the genotypes coded as 1,2,3 for each animal. Because of computational limitations, the size of the data set was further reduced by selecting SNPs based on their connectivity, which is the sum of the connection strengths of a particular SNP with all other SNPs. Genes with a high connectivity, also called hubgenes, are thought to be biologically important and therefore, only the top 2500 SNPs were selected (normalized connectivity > 0.12). To pursue scale-free topology, a power γ was chosen in such a way that the *R*^2^ (the scale-free topology index) approaches one. A power γ of 5 resulted in an *R*^2^ of 0.88, which was used to create an adjacency matrix by calculating the Pearson's correlations among SNPs and raising this to the power γ of 5. The network was constructed based on the topological overlap measure (TOM) between SNPs, where a high TOM represents a high share of neighbors between a pair of SNPs, and consequently, a low TOM represents a low share of neighbors between a pair of SNPs. Based on the TOM, clusters of highly interconnected SNPs were detected, called modules, using the Dynamic Tree Cutting algorithm (Langfelder et al., 2008). Modules were selected for downstream analysis based on their Module-Trait Relation (MTR), which is calculated by correlating the module eigenSNP (the first principal component, explaining most of the variance in the module) with the OI and other OOR traits. *P*-values were represented by the Student asymptotic *p*-value for the given correlations. Other OOR traits were represented by the estimated breeding values, previously calculated (Kogelman et al., 2013). Modules with a significant correlation (*P*-value < 0.001) with the OI and an MTR > 0.4 with at one other OOR trait were selected for pathway analysis.

#### Differential wiring of SNPs in obesity (DW networks)

It is expected that biologically important pathways related to the trait under investigation, will show a different activity pattern between the two extreme groups. In other words, a pathway could be induced in the case-group, while repressed in the control-group. Highly interconnected SNPs, and consequently their mapped genes, are called hub genes, which are potentially biologically relevant genes. Therefore, we investigated which SNPs were DW (connected) between the lean and obese animals. The interconnectivity is represented by the sum of correlations of a particular SNP with all other SNPs within a (obese or lean) sub-network. First, the 50 most extreme lean animals and the 50 most extreme obese animals were selected based on the OI. Based on the same selected SNPs (*n* = 40,194) as in the normal network construction the connectivity of all SNPs in the lean (k_lean) and obese (k_obese) subnetwork was calculated. Then, the differential wiring or connectivity (k_diff) was calculated by subtracting k_obese from k_lean. This resulted in a positive k_diff for SNPs that had high interconnectivity in the lean subnetwork, but low interconnectivity in the obese subnetwork. Subsequently, negative values for k_diff were found for SNPs that had high interconnectivity in the obese subnetwork, but low interconnectivity in the lean subnetwork. SNPs with an absolute k_diff above 0.6 were selected for further investigation.

### Pathway analysis

#### SNP selection and gene detection

SNPs that were cross-validated as being highly significant by both GWA and LDLA study and those SNPs that were present in the modules as being highly associated with the OI from WISH network construction were all selected for further pathway profiling analyses. Genes mapping to the detected SNPs were obtained using Biomart (Ensembl v73) (Haider et al., 2009). All identified genes were used for pathway analysis. Secondly, to cover the promoter region of genes, which lay outside positions covered by Biomart, we used the NCBI2R R-package (Melville and Melville, 2012) (available at http://cran.r-project.org/web/packages/NCBI2R/index.html) which uses a list of SNPs as an input and gives (if present) the genes and their annotation in the indicated region. We used a flanking distance of 20 kB, as the average gene size is 30 kB and we used an extra 5 kB to cover the promoter region.

#### Pathway detection

Genes located within 20 kb of SNPs identified via GWA and WISH network analyses (modules) were used for gene enrichment analysis of biological pathways in order to identify the potential biological relevance of detected SNPs. Since pathway analysis is very dependent on the databases used for biological annotation, we used different tools that leverage information available in different publically available tools to complement each other: NCBI2R (available at http://cran.r-project.org/web/packages/NCBI2R/index.html), Gene Ontology Enrichment Software Toolkit (GOEAST) (Zheng and Wang, 2008) and GeneNetwork (http://www.genenetwork.nl).

Using the R-package NCBI2R, pathways in the identified genes were detected using the *GetPathways()* function. The same function was used on all genes present in and around (flanking distance = 20 Kb) the SNPs which passed QC, as a reference pathway set. The significance level of present pathways was calculated using the Fisher's exact test and multiple-testing correction was applied using the Bonferroni-correction. Secondly, GOEAST was used to identify overrepresented GO terms among the identified genes. The *Gene Batch* tool in GOEAST was used to import the gene symbols and to identify significantly overrepresented GO terms and corresponding pathways were visualized (Zheng and Wang, 2008). Thirdly, GeneNetwork (http://www.genenetwork.nl) was used to identify overrepresented GO terms, KEGG pathways, phenotypes, and tissues. GeneNetwork is constructed using human, mouse, and rat expression data, to predict gene functions against known pathways and gene sets in various biological databases. Overrepresentation of GO-terms and pathways was tested within the GeneNetwork tool, using the Mann–Whitney U test, and *P*-values were afterwards corrected for multiple testing using the Bonferroni correction.

## Author contributions

Haja N. Kadarmideen was the project leader and contributed to designing quantitative- and systems genetics analyses including single SNP association methods, obesity index, network construction and pathway profiling approaches, and supervised Lisette J. A. Kogelman in these analyses. Merete Fredholm contributed to designing the F2 resource population and supervised collection of biological material and phenotypic measurements on all pigs. Lisette J. A. Kogelman analyzed all the data. The LDLA analysis was performed by Sameer D. Pant. Lisette J. A. Kogelman wrote the first draft of the manuscript. All authors wrote, read, and approved the final version of the manuscript.

### Conflict of interest statement

The authors declare that the research was conducted in the absence of any commercial or financial relationships that could be construed as a potential conflict of interest.
